# Recent Advances in Treating Psychiatric Disorders in Children and Adolescents: From Theory to Practice

**DOI:** 10.3390/jcm13237489

**Published:** 2024-12-09

**Authors:** Pietro Muratori, Valentina Levantini

**Affiliations:** 1 IRCCS Fondazione Stella Maris, Scientific Institute of Child Neurology and Psychiatry, 56018 Pisa, Italy; 2 Department of Languages and Literatures, Communication, Education and Society, University of Udine, 33100 Udine, Italy; valentina.levantini@hotmail.it

We are pleased to introduce this Special Issue of the *Journal of Clinical Medicine*, dedicated to “(Recent Advances) Treating Psychiatric Disorders in Children and Adolescents: From Theory to Practice”, which addresses one of the most critical topics in mental health nowadays. Childhood and adolescence are crucial developmental stages where mental health challenges can profoundly shape one’s life trajectory [1]. Studies have consistently shown that mental health issues often arise during these stages [2,3], and their negative consequences may persist into adulthood [4,5]. This highlights the utmost importance of further research to identify novel intervention targets and develop innovative treatments [6].

Over the past few decades, the understanding of psychiatric disorders in younger populations has advanced significantly [7]. This Special Issue brings together recent advancements and clinical insights, reflecting the latest developments in both research and applied practice. Our contributors share diverse perspectives on emerging methodologies and treatment frameworks that hold promise for improving outcomes for youths and their families.

This Special Issue covers a broad spectrum of conditions, including externalizing problems, specific learning disabilities, fetal alcoholic syndrome, tic disorders, and personality disorders. As readers explore these pages, they will encounter compelling studies and reviews that provide an insightful perspective on the multifaceted journey from research and theoretical advances to practical application.

Some of the studies included in this Special Issue aim to shed light on the unique factors at play in specific populations. Grossi et al. (Contribution 1) explored the link between psychopathic traits, social dominance orientation, externalizing problems, and prosocial behavior in adolescents, providing valuable evidence on the correlates of psychopathic traits in youth with Oppositional Defiant Disorder or Conduct Disorder. As youths with psychopathic traits typically exhibit more severe symptoms [8] and are less responsive to traditional intervention models [9], the findings described by Grossi et al. are particularly relevant, offering valuable information for treatment efforts.

Fetal alcohol syndrome can lead to severe neurodevelopmental impairment, congenital anomalies, and/or poor growth [10]. Despite being one of the leading causes of birth defects and developmental disability, its prevalence exceeds 1% [11], suggesting that more research is clearly needed. In this context, Grzywacz et al. (Contribution 2) investigate environmental and biological factors related to fetal alcohol syndrome, highlighting the maternal drinking patterns that might lead to the most severe outcomes. Additionally, Cristofani et al. (Contribution 3) explored the emotional–behavioral phenotype of children and adolescents with Specific Learning Disabilities (SLD) and evaluated the mediating role of background and cognitive functioning, emphasizing the complexity of the interaction between cognitive, learning, and emotional–behavioral phenotypes. The authors further highlighted the need for a specific evaluation of emotional and behavioral difficulties in children with SLD [12,13]. 

Other articles offered an evidence-based route for translating theoretical frameworks into practical treatment solutions, reflecting the deep commitment of clinicians and researchers working together to improve youths’ well-being. In this regard, Boxmeyer et al. (Contribution 4) integrated mindful activities into the Coping Power Program [14,15], leading to a promising preventive intervention—the Mindful Coping Power—with long-lasting effects on embodied awareness, self-regulation, stress physiology, and behavioral outcomes in at-risk children.

Finally, two manuscripts eloquently reviewed the extant literature on particularly relevant topics. Malaty et al. (Contribution 5) summarized findings on Functional Neurological Disorder and Tourette syndrome, providing helpful advice for diagnosis and intervention that might support clinicians, given the increasing requests for evaluations of these conditions [16]. In contrast, Bourvis et al. (Contribution 6) described the current understanding of Borderline Personality Disorder in adolescents, which is characterized by more severe symptoms and outcomes [17], and how to translate these pieces of information into effective care strategies.

Overall, this Special Issue stands as a testament to the progress made thus far, while also emphasizing the need for continued exploration to identify novel ways to better tailor treatments and meet the needs of children and adolescents. At the same time, future research is essential to overcome systemic obstacles, ensuring access to quality care and fostering resilience and well-being beyond the clinical setting.

We extend our gratitude to all the authors who contributed to this Special Issue and demonstrated outstanding commitment to advancing this important research topic. We hope this Special Issue serves as a valuable resource and stimulates further research, dialog, and innovation in child and adolescent psychiatry.
